# Reliable Detection of Myocardial Ischemia Using Machine Learning Based on Temporal-Spatial Characteristics of Electrocardiogram and Vectorcardiogram

**DOI:** 10.3389/fphys.2022.854191

**Published:** 2022-05-30

**Authors:** Xiaoye Zhao, Jucheng Zhang, Yinglan Gong, Lihua Xu, Haipeng Liu, Shujun Wei, Yuan Wu, Ganhua Cha, Haicheng Wei, Jiandong Mao, Ling Xia

**Affiliations:** ^1^ School of Instrument Science and Opto-Electronic Engineering, Hefei University of Technology, Hefei, China; ^2^ School of Electrical and Information Engineering, North Minzu University, Yinchuan, China; ^3^ Key Laboratory of Atmospheric Environment Remote Sensing of Ningxia, Yinchuan, China; ^4^ Department of Clinical Engineering, The Second Affiliated Hospital, Zhejiang University School of Medicine, Hangzhou, China; ^5^ Hangzhou Maixin Technology Co., Ltd., Hangzhou, China; ^6^ Institute of Wenzhou, Zhejiang University, Wenzhou, China; ^7^ Hangzhou Linghua Biotech Ltd., Hangzhou, China; ^8^ Research Centre for Intelligent Healthcare, Coventry University, Coventry, United Kingdom; ^9^ Department of Cardiology, Ningxia Hui Autonomous Region People’s Hospital, Yinchuan, China; ^10^ Key Laboratory for Biomedical Engineering of Ministry of Education, Institute of Biomedical Engineering, Zhejiang University, Hangzhou, China

**Keywords:** myocardial ischemia, vectorcardiogram (VCG), sample entropy (SampEn), Lyapunov index, support vector machine (SVM)

## Abstract

**Background:** Myocardial ischemia is a common early symptom of cardiovascular disease (CVD). Reliable detection of myocardial ischemia using computer-aided analysis of electrocardiograms (ECG) provides an important reference for early diagnosis of CVD. The vectorcardiogram (VCG) could improve the performance of ECG-based myocardial ischemia detection by affording temporal-spatial characteristics related to myocardial ischemia and capturing subtle changes in ST-T segment in continuous cardiac cycles. We aim to investigate if the combination of ECG and VCG could improve the performance of machine learning algorithms in automatic myocardial ischemia detection.

**Methods:** The ST-T segments of 20-second, 12-lead ECGs, and VCGs were extracted from 377 patients with myocardial ischemia and 52 healthy controls. Then, sample entropy (*SampEn*, of 12 ECG leads and of three VCG leads), spatial heterogeneity index (*SHI*, of VCG) and temporal heterogeneity index (*THI*, of VCG) are calculated. Using a grid search, four *SampEn* and two features are selected as input signal features for ECG-only and VCG-only models based on support vector machine (SVM), respectively. Similarly, three features (*S*_*I*_, *THI*, and *SHI*, where *S*_*I*_ is the *SampEn* of lead I) are further selected for the ECG + VCG model. 5-fold cross validation was used to assess the performance of ECG-only, VCG-only, and ECG + VCG models. To fully evaluate the algorithmic generalization ability, the model with the best performance was selected and tested on a third independent dataset of 148 patients with myocardial ischemia and 52 healthy controls.

**Results:** The ECG + VCG model with three features (*S*_*I*_,*THI*, and *SHI*) yields better classifying results than ECG-only and VCG-only models with the average accuracy of 0.903, sensitivity of 0.903, specificity of 0.905, F1 score of 0.942, and AUC of 0.904, which shows better performance with fewer features compared with existing works. On the third independent dataset, the testing showed an AUC of 0.814.

**Conclusion:** The SVM algorithm based on the ECG + VCG model could reliably detect myocardial ischemia, providing a potential tool to assist cardiologists in the early diagnosis of CVD in routine screening during primary care services.

## 1. Introduction

Myocardial ischemia is a condition in which the perfusion of heart muscle is insufficient due to an obstructive plaque, coronary artery spasm, or coronary microvascular dysfunction (Liu H. et al., 2020; Paolo Severino, 2020). Myocardial ischemia can lead to cardiovascular events including acute myocardial infarction death and sudden cardiac death (SCD) (Moran et al., 2014). It accounts for 16% of the world’s total deaths and has been listed as a leading cause of mortality by the World Health Organization (WHO), with the prevalence rate per 100,000 population supposed to increase from 1,655 to 1845 by the year 2030 (Khan et al., 2020).

Clinically, the gold standard of myocardial ischemia diagnosis is the invasive measurement of fractional flow reserve (FFR) and index of microcirculatory resistance (IMR) using a coronary guidewire (Kaski et al., 2018; Geng et al., 2022). Clinical imaging techniques have also been applied to myocardial ischemia detection, including invasive coronary angiography, computed tomography (CT) coronary angiography, nuclear myocardial perfusion imaging, and cardiac magnetic resonance (CMR) (Kaski et al., 2018). However, due to their invasiveness, radiation, high cost, and complicated operations that need special training, the guidewire measurements and imaging modalities are usually applied to patients with existing ischemic symptoms. There is an increasing clinical need for a non-invasive, low-cost, and convenient method to achieve early detection of myocardial ischemia.

**FIGURE 1:**
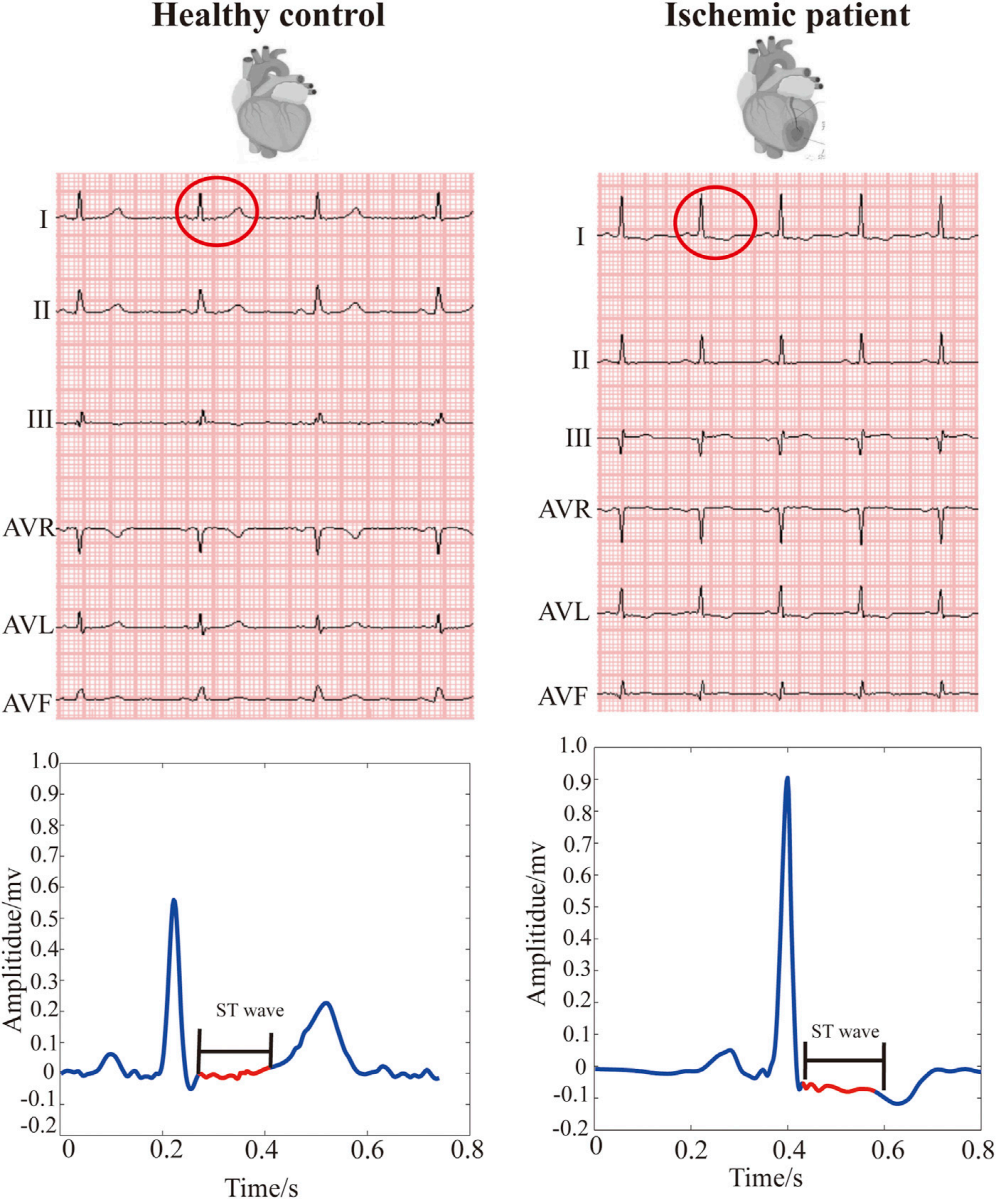
Ischemia-related changes in ST-wave (ST-segment). **(A)** ST wave from a healthy control. **(B)** ST wave from a patient with myocardial ischemia. Red line presents ST wave in ECG.

An electrocardiogram (ECG) is a non-invasive and low-cost method to detect cardiac electrophysiology. Myocardial ischemia can lead to specific changes in the ECG waveform. As shown in Figure 1, the earliest manifestations of myocardial ischemia in the ECG waveform include transient ST-elevation or ST-depression and T-wave changes (Thygesen et al., 2012). These typical changes in T wave and ST segment are commonly used as indicators of myocardial ischemia. At present, ECG is the first-line diagnostic tool in the assessment of patients with suspected myocardial ischemia (Fihn et al., 2014). Due to the difficulty of capturing subtle changes in ST-T segments, the sensitivity of manual inspection in diagnosing myocardial ischemia is only about 60% (Dehnavi et al., 2011; Stefan Weber et al., 2014). Even patients with severe coronary stenosis may have no observable ECG changes (Barstow et al., 2017).

To overcome the limitations of manual inspection, computer-aided diagnostic frameworks based on myocardial ischemia-relevant ECG features have been proposed (Alizadehsani et al., 2019).To improve the sensitivity in myocardial ischemia classification, most algorithms used for myocardial ischemia classification and diagnosis focus on the feature extraction from heart rate variability (HRV) (Goldenberg et al., 2019), beat-based techniques (Acharya et al., 2016) or frame-based schemes (a few consecutive beats) (Sharma and Sunkaria, 2017; Braun et al., 2020; Butun et al., 2020).

In particular, vectorcardiogram (VCG) can further empower the ECG-based automatic detection of myocardial ischemia. VCG is a special form of ECG and can be mathematically synthesized from standard 12-lead ECG. The VCG consists of three orthonormal leads (X, Y, and Z), reflecting cardiac electric activity in the frontal, horizontal, and sagittal planes (Grishman et al., 1951). Compared with the standard 12-lead ECG, VCG represents both the magnitude and spatial information of heart activity (Burger et al., 1956). Hence, VCG provides higher sensitivity than ECG in diagnosing myocardial ischemia, e.g., 70% using manual inspection (Dehnavi et al., 2011), without sacrificing specificity (Hurd et al., 1981; Sascha et al., 2021). The combination of ECG and VCG could achieve even better performance (Lee et al., 1968; Correa et al., 2016). Authors demonstrated that combination of ECG and ECG-reconstructed VCG can achieve comparable performance to the combination of ECG and measured VCG in detecting myocardial ischemia (Bemmel et al., 1992; Kors et al., 1992). Therefore, VCG-enhanced automatic early detection of myocardial ischemia has gained increasing popularity (Ansari et al., 2017).

VCG features extracted automatically are employed to detect ischemic beats (Häggmark et al., 2008; Aranda Hernandez et al., 2018; Braun et al., 2020; Chuang et al., 2020) or frames (Rahul et al., 2021), discriminate myocardial ischemia (Deng et al., 2017), localize the culprit infarct-related arteries in acute myocardial infarction (Le et al., 2020), and screen for the presence of scar tissue in the myocardium (Uyen Chau et al., 2018).

Deep features can be extracted from ECG or VCG in heart beats or frames using various mathematical transforms (Acharya et al., 2016). The number of features is highly diverse in existing studies. 12 nonlinear ECG features (Liu J. et al., 2020), 72 multiscale energy and eigenspace ECG features (Sharma et al., 2015), 288 ECG features in time, frequency, nonlinear, and entropy domains (Liu et al., 2019), 7 (Correa et al., 2013) and 52 features of 3-lead ECG (Chuang et al., 2020), 290 multidimensional parameters of 5-lead VCG (Braun et al., 2020), 22 features of 6-lead VCG (Dehnavi et al., 2011), and 322 pseudo-VCG features (Aranda Hernandez et al., 2018). These features are fed into machine learning (ML) models (Aranda Hernandez et al., 2018; Chuang et al., 2020) to develop computer-aided diagnostic models of myocardial ischemia, achieving higher efficiency and accuracy than manual inspection.

Existing computer-aided approaches for myocardial ischemia detection suffer from some limitations. Firstly, there are few studies on the combination of ECG and VCG in detecting myocardial ischemia. It has been demonstrated that the models with both VCG and ECG features yielded the highest performance, followed by the VCG-only and ECG-only models, while the details of the algorithms were not disclosed (Bemmel et al., 1992; Kors et al., 1992). In 2021, Pollard *et al.* drew a similar conclusion using eight different models based on global electrical heterogeneity extracted from ECGs and VCGs (Pollard et al., 2021). Secondly, the high-dimensional features lead to an oversaturation of small datasets and a high computational burden. Some features are correlated or insignificant for the classification (Pollard et al., 2021). The minimization of the feature set is essential to enhance the recognition capabilities of a model. Thirdly, beat- or frame-based approaches are usually used, which results in the incapacity to detect the subtle ST-T changes in continuous cardiac cycles and oversampling. Oversampling may weaken the model’s feasibility and adaptability (Fu et al., 2020). Longer ECG records can avoid the oversample and enhance the total scheme efficiency and overall accuracy for the myocardial ischemia diagnosis algorithm (Li et al., 2021).

To overcome the above-mentioned limitations, we propose a novel algorithm for myocardial ischemia detection based on three selected features extracted from ST-T segments of 20 s, 12-lead ECGs, and derived 3-lead VCGs. Three support vector machine (SVM) models fed with different signal features (ECG-only, VCG-only, and ECG + VCG) are trained and tested. The model with the best performance was selected as a potential approach towards accurate, non-invasive, and low-cost detection of myocardial ischemia.

## 2. Materials and Methods

**FIGURE 2:**
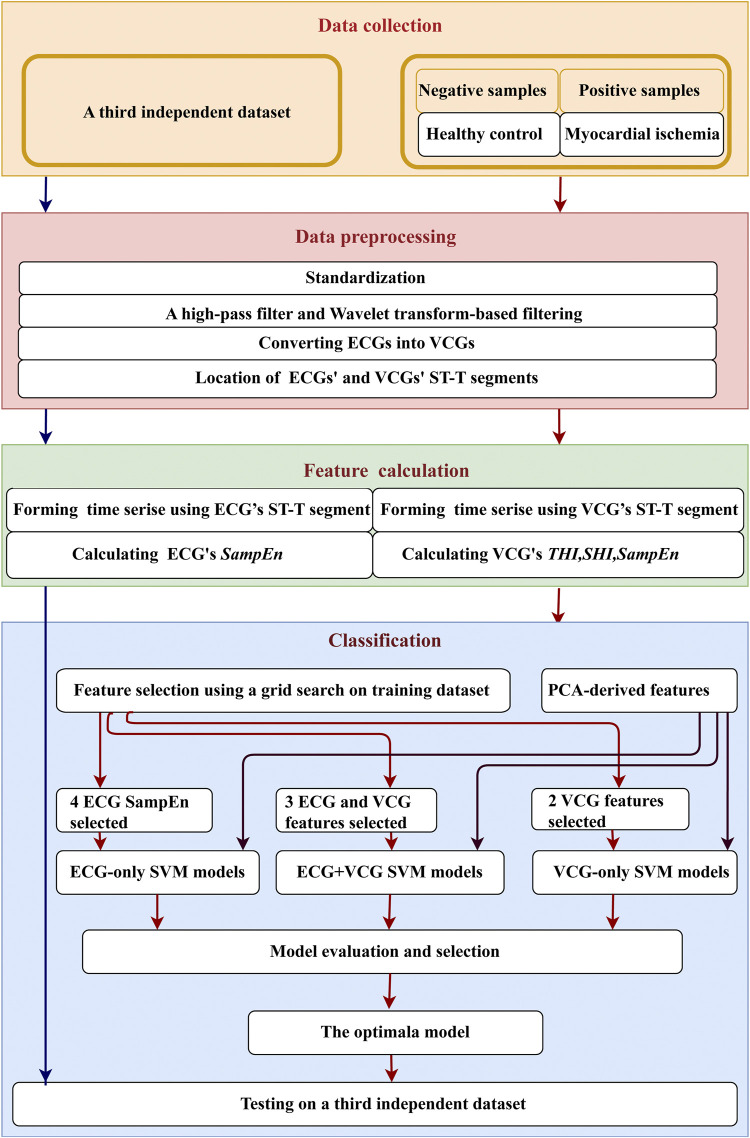
System framework.

As shown in Figure 2, work consists of four parts: data collection, preprocessing, feature calculation, and classification. Firstly, 20-second (20 s), 12-lead ECGs were collected and converted into 3-lead VCGs. The onset of ST-wave and offset of T-wave were marked on the ECGs and VCGs. Then, multi-domain characteristics analysis was performed to extract the features, including sample entropy (*SampEn*) from ECGs’ and VCGs’ ST-T segments, as well as spatial heterogeneity index (*SHI*) and temporal heterogeneity index (*THI*) from VCGs’ ST-T segments. Subsequently, the most effective features were selected from the training dataset, combined (i.e., ECG-only, VCG-only, ECG + VCG), and deployed in support vector machine (SVM) models for myocardial ischemia identification. To validate the feature selection results, the classification performance of the selected features was compared with that of principal component analysis (PCA)-derived features. With 5-fold cross validation based on clinical diagnosis, the results of three SVM models (ECG-only, VCG-only, and ECG + VCG) were comprehensively evaluated and compared to investigate if VCG features can improve the accuracy of myocardial ischemia detection. Finally, the final selected model was tested on a third independent dataset.

### 2.1 Data Collection

The 10-second (10 s) ECG is common in clinical practice, whilst longer ECGs can improve the total scheme efficiency and overall accuracy of myocardial ischemia detection algorithms (Li et al., 2021). However, it is difficult for some patients to stay in the supine posture for long period to get high-quality ECGs. Consequently, 20sec segments are adopted in this work.

#### 2.1.1 Datasets for Training and Validation

In this study, clinical data was collected from 429 subjects in two cohorts. The data of 52 healthy controls (age: 43 ± 17 years; 41 males and 11 females) were from the Physikalisch-Technische Bundesanstalt (PTB) diagnostic ECG database (https://www.physionet.org/content/ptbdb/1.0.0/) (Bousseljot et al., 1995; Goldberger et al., 2000). The ECGs in this collection were obtained using a non-commercial, PTB prototype recorder with 16-bit resolution at a resolution of 0.5 μV/LSB and a sampling frequency of 1,000 Hz.

From November 2014 to November 2015, the data of 377 patients with myocardial ischemia were obtained from FuWai Hospital, Beijing, China, with approval from the local ethics committee for sharing and analyzing retrospective anonymized patient data with informed consent form waived. Inclusion criteria were suspected patients with coronary artery disease (CAD) for coronary angiography with simultaneous ECG records. The exclusion criteria were as follows:

1) The presence of heart diseases such as heart valve disease, congestive heart failure, pulmonary arterial hypertension, or left ventricular hypertrophy;
2) The presence of bundle branch blocks, as well as of non sinus or paced rhythm.

The hospital diagnosis of myocardial ischemia, which was used as the ground truth, was made by professional cardiologists based on comprehensive analysis of clinical data and the following positive indicators:

1) Suggestive clinical history and clinical examination;
2) Presence of coronary stenosis of >50%.

The 20 s, 12-lead resting ECGs were recorded using a commercially available ECG device (Mindray Bene-Heart R12, Shenzhen, China) with 16 bit precision at a resolution of 1 μV/LSB and a sampling frequency of 1,000 Hz. The characteristics of 377 ischemic patients are presented in Table 1.

**TABLE 1:** Clinical characteristic of ischemic patients

Characteristics	Values^*^
Age (years)	58 ± 10
Female	91/377
Chest pain	228/377
Dyspnea	196/377
Heart rate (bpm)	70 ± 10
Ejection fraction (%)	62 ± 6
Left ventricular end diastolic diameter (mm)	48 ± 5
Systolic blood pressure (mmHg)	129 ± 16
Diastolic blood Pressure (mmHg)	79 ± 12
Smoker	225/377
Family history of CAD	19/377

* The continuous values were provided as mean ± SD, for normally distributed data while the categorical data was presented as numbers and percentages.

Finally, the 20 s, 12-lead ECGs from two groups (377 ischemic patients and 52 healthy controls, as positive and negative samples, respectively) were sampled at 1,000 Hz frequency with 16-bit precision. Figure 1 shows the ECGs of two subjects from the ischemic patient and control groups.

#### 2.1.2 A Third Independent Database for Testing

To fully evaluate algorithmic generalization ability, a third independent dataset was implemented for testing. The data was collected from 200 subjects in two cohorts. Regarding positive samples, 148 20 s ECGs were collected from 148 patients with myocardial infarction (age: 60 ± 11 years, 108 males and 40 females, 67 smokers, systolic blood pressure: 121 ± 19 mmHg, and diastolic blood pressure: 74 ± 13 mmHg) of the PTB diagnostic ECG database with 16 bit precision at a resolution of 0.5 μV/LSB and a sampling frequency of 1,000 Hz (Goldberger et al., 2000).

As for negative samples, 52 20 s ECGs were randomly collected from 52 healthy controls (age: 37 ± 15 years, 18 males and 34 females) of the China Physiological Signal Challenge in 2018 (CPSC 2018) database (http://2018.icbeb.org/Challenge.html) with 16 bit precision at a resolution of 1 μV/LSB and a sampling frequency of 500 Hz (Perez Alday et al., 2021; Reyna Ma, 2021; Reyna Ma, 2022). Then, the 52 20 s ECGs were resampled at 1,000 Hz.

### 2.2 Data Preprocessing

Firstly, the baseline drift and low-frequency fluctuations (e.g., respiratory movements) were removed using a high-pass Butterworth filter at 0.67 Hz. The cutoff frequency setting in high-pass filtering could significantly influence the morphology of ST segments. The cutoff frequency of 0.67 Hz is recommended for diagnostic purposes since it could remove baseline drifts with no obvious distortion of ST segments (Roger Abächerli and Schmid, 2009; Luo and Johnston, 2010).

Subsequently, high-frequency noises, including power-line interference (50/60 Hz) and electromyogram noise (10–230 Hz) (Yazdani et al., 2017), should be removed since they can affect the localization of ST-segments in ECG waves and affect the detection of myocardial ischemia (Christov et al., 2017). Band-pass filtering is widely used in ECG signal processing for eliminating high-frequency noise and performs well in R-peak detection. Regarding two commonly used cutoff frequencies (i.e., 40 and 150 Hz) in low-pass filtering of ECGs, 40 Hz could effectively eliminate the high-frequency noises but lead to the elevation of J-point, i.e., the junction between QRS termination and ST-segment onset (Nakagawa et al., 2014; Christov et al., 2017), resulting in inaccuracy of the onset of the ST segment, whereas 150 Hz could overcome this problem but cause a high level of residual noise (Ricciardi et al., 2016; Christov et al., 2017). As compared with a band-pass filter, the discrete wavelet transform could perform better in terms of eliminating high-frequency noise and keeping the morphology feature points of the ECG signal (Addison, 2005; Singh and Pradhan, 2018; Chen et al., 2020), as illustrated in Supplementary Figure S1. Therefore, high-frequency noise was removed using discrete wavelet transform and wavelet thresholding (Kumar et al., 2021). Coif4 was utilized as a wavelet basis function to decompose noise-containing ECGs into four layers. The denoized ECGs were reconstructed using the inverse of the discrete wavelet transform followed by the elimination of noise by an adaptive threshold.

Next, 20 s,12-lead ECGs were standardized based on 25 mm/s using a gain setting of 10 mm/mV (Kossmann et al., 1967) and then transformed into VCGs (Jaros et al., 2019):

$$\{\begin{array}{c} V_{x}=0.38I-0.07II-0.13V1+0.05V2-0.01V3+0.14V4+0.06V5+0.54V6\\ V_{y}=-0.07I+0.93II+0.06V1-0.02V2-0.05V3+0.06V4-0.17V5+0.13V6\\ V_{z}=0.11I-0.23II-0.43V1-0.06V2-0.14V3-0.20V4-0.11V5+0.31V6 \end{array}.$$ (1)

**FIGURE 3:**
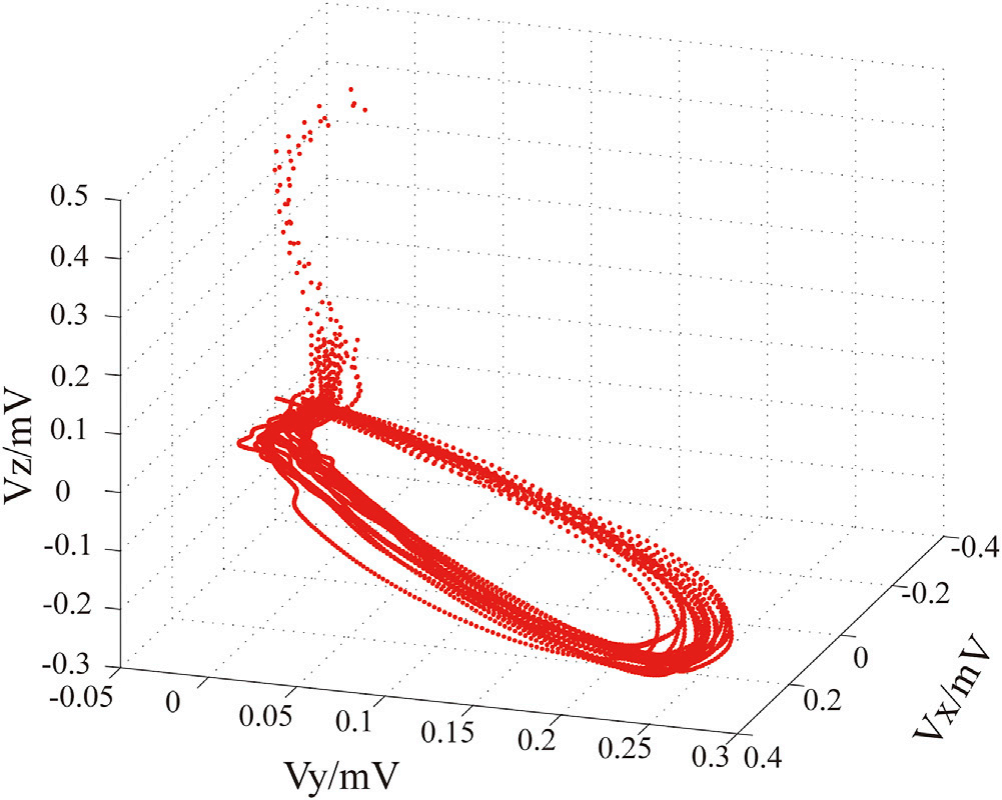
Three-dimensional VCGs’ ST-T segments derived from 20sec, 12-lead ECGs.

Finally, the ST-T segments of ECGs and of VCGs were detected employing a hybrid approach (Song et al., 2012). The three-dimensional (3D) ST-T segments of VCGs are shown in Figure 3.

### 2.3 Feature Extraction

Feature extraction is the process of revealing hidden ischemia-related characteristics from ECGs and VCGs, which lays the groundwork for detecting myocardial ischemia. In our proposed scheme, features were extracted from ST-T segments in the entropy domain, frequency domain, and Lyapunov index, separately.

#### 2.3.1 *SampEn*

For detecting myocardial ischemia, various entropies calculated from HRV (Udhayakumar et al., 2019), ST segments (Rabbani et al., 2011; Wei et al., 2012) or filtered 12-lead ECGs (Liu et al., 2019) have been used. To quantitatively evaluate the complexity of physiological time-series and diagnose diseases, Richman and Moorman refined the approximate entropy algorithm and introduced sample entropy (*SampEn*) by excluding the self-matching of templates’ data length (Richman and Moorman, 2000). *SampEn*, defined as the negative natural logarithm of a conditional probability, was employed in this study, since it is largely independent of record length and enables more consistent calculation results compared with approximate entropy (Richman and Moorman, 2000). Given that two data sequences are similar for m points, they remain similar at the next point, within a tolerance “*r*” that represents a fraction of the series standard deviation. *SampEn* is positively related to the signal complexity of each ECG lead. Overall, the *SampEn* obtained from the ST segment of healthy controls was less than that of ischemic patients (Rabbani et al., 2011). Therefore, *SampEn* may be an early indicator of myocardial ischemia.

In our method, *SampEn* values were calculated from the ST-T segments of ECGs ( $S_{i},i=I,II,III,AVR,AVL,AVF,V1,V2,V3,V4,V5,V6$ ) and VCGs ( $S_{i},i=V_{x},V_{y},V_{z}$ ) based on existing methods (Richman and Moorman, 2000; Antonio Molina-Picó et al., 2011; Marwaha and Sunkaria, 2016). The major steps are as follows: Firstly, for each ECG or VCG lead, its time series, i.e., {x(n)}=x(1),x(2),⋯,x(N) was formed by splicing beat-to-beat ST-T segments followed by standardization: $x=\frac{x-\mu }{\sigma }$ . where μ and σ are mean value and the standard deviation of the whole time series. N is the length of the time series.Then, the vectors $X_{m}\left(1\right),\cdots ,X_{m}\left(N-m+1\right)$ and $X_{m+1}\left(1\right),\cdots ,X_{m+1}\left(N-m\right)$ with a dimension of m and m+1 were formed, respectively. Here $X_{m}\left(j\right)=\left\{x\left(j\right),x\left(j+1\right),\cdots ,x\left(j+m-1\right)\right\},1\leq j\leq N-m+1$ and $X_{m+1}\left(j\right)=\left\{x\left(j\right),x\left(j+1\right),\cdots ,x\left(j+m\right)\right\},1\leq j\leq N-m$ .

Subsequently, the distance between $X_{m}\left(j\right)$ and X(z)m was defined as:

$$d\left\{X_{m}\left(j\right),X_{m}\left(z\right)\right\}=\underset{0\leq k\leq m-1}{\max}\left(\left|x\left(j+k\right)-x\left(z+k\right)\right|\right),1\leq z,j\leq N-\mathrm{m+1},z\neq j.$$ (2)

$A_{j}\left(m,r\right)$ and $A_{j}\left(m+1,r\right)$ were defined as:

$$A_{j}\left(m,r\right)=\frac{n_{j}\left(m,r\right)}{N-m-1},j=1,\cdots ,N-m+1,$$ (3)

$$A_{j}\left(m+1,r\right)=\frac{n_{j}\left(m-1,r\right)}{N-m-1},j=1,\cdots ,N-m.$$ (4)

Here $n_{j}\left(m,r\right)$ presents the number of vectors $X_{m}\left(j\right)$ within r of vector $X_{m}\left(z\right)$ : $d\left(X_{m}\left(j\right),X_{m}\left(z\right)\right)<r$ . *r* is the error tolerance range of similar regions and is recommended between 0.1 and 0.25 times the standard deviation of time series. $n_{j}\left(m+1,r\right)$ presents the number of vectors $X_{m+1}\left(j\right)$ within r of vector $X_{m+1}\left(z\right)$ : $d\left(X_{m+1}\left(j\right),X_{m+1}\left(z\right)\right)<r$ , z ranges from 1 to N−m , and z≠j .

$$\varphi \left(m,r\right)=\frac{1}{N-m}\sum_{j=1}^{N-m}A_{j}\left(m,r\right),$$ (5)

$$\varphi \left(m+1,r\right)=\frac{1}{N-m}\sum_{j=1}^{N-m}A_{j}\left(m+1,r\right).$$ (6)

Here φ(m,r) and φ(m+1,r) are the probability that two sequences match at m and m+1 points, respectively.

Finally, *SampEn* was calculated as follows:

$$SampEn\left(N,m,r\right)=-\ln\frac{\varphi \left(m+1,r\right)}{\varphi \left(m,r\right)},$$ (7)

where the parameters *r* and *m* were set to the best value (Richman and Moorman, 2000; Marwaha and Sunkaria, 2016) of r=0.1,m=2 .

#### 2.3.2 *SHI* and *THI*

The spatial heterogeneity index (*SHI*) and temporal heterogeneity index (*THI*) reflect the spatial and temporal heterogeneity of 3D trajectory, respectively (Deng et al., 2017). *SHI* (Deng et al., 2017) was calculated based upon the Lyapunov index to describe the spatial characteristics of VCGs’ ST-T segments, as described in Eq. 8.

$$SHI=\frac{1}{N}\sum_{n=1}^{N}\ln\left(\frac{d_{n2}}{d_{n1}}\right),$$ (8)

where N presents the length of VCGs’ ST-T segments, $d_{n1}$ is the distance between the n^th^ data point and its nearest data points, and $d_{n2}$ denotes the distance between the n^th^ data points and its nearest data points after 10 steps. *THI* (Deng et al., 2017) was calculated to reveal the temporal characteristics of VCGs’ ST-T segments:

$$f_{i}\left(w\right)=abs\left(F\left(Vs_{i}\right)\right),i=x,y,z,$$ (9)

where F is the Fourier transform, and *Vs*_*i*_ represents the VCGs’ ST-T segments.

Then, the $f_{i}\left(w\right)$ was fitted as an exponential function with an exponent $\lambda_{i}$ .

$$\gamma_{i}=\arg\min_{\lambda_{i}}\left|f_{i}\left(w\right)-f_{i}\left(w\right)\exp\left(-0.001\lambda_{i}\right)\right|,i=x,y,z,\lambda_{i}=1:length\left(f_{i}\left(w\right)\right).$$ (10)

Finally, *THI* was calculated as follows:

$$THI=\sqrt{\frac{\sum_{i=1}^{3}\gamma_{i}^{2}}{3}}.$$ (11)

### 2.4 Myocardial Ischemia Detection Using SVM

#### 2.4.1 The Proposed Models

The SVM model is one of the most frequently utilized ML models for cardiovascular disease detection (Alizadehsani et al., 2019) and is constructed by projecting input vectors into a higher dimensional space via a kernel function and capturing the decision boundary (formally known as a hyperplane) with the maximum margin between different classes. It has the advantages of reducing empirical errors, preserving the complexity level of the mapping function, and ensuring better performance. Thus, an SVM model with the Gaussian radial basis function (RBF) kernel was employed in this study to distinguish between healthy controls and ischemic patients.

The extracted features were combined into vectors and fed into the SVM model to classify the subjects (i.e., healthy control vs. ischemic patients). For the model design, the ECG-only model presented the SVM framework fed with ECGs’ *SampEn* only. Similarly, the VCG-only model employed only the VCGs’ features in the SVM framework. Finally, the ECG + VCG model presented the SVM framework utilizing selected ECG and VCG features.

#### 2.4.2 Feature Selection

Feature selection plays a critical role in improving the performance of classification algorithms by identifying relevant features and discarding irrelevant ones. Generally, a subset of available features includes all relevant features, whereas the remaining irrelevant features do not contribute to the classification (Urbanowicz et al., 2018).

**FIGURE 4:**
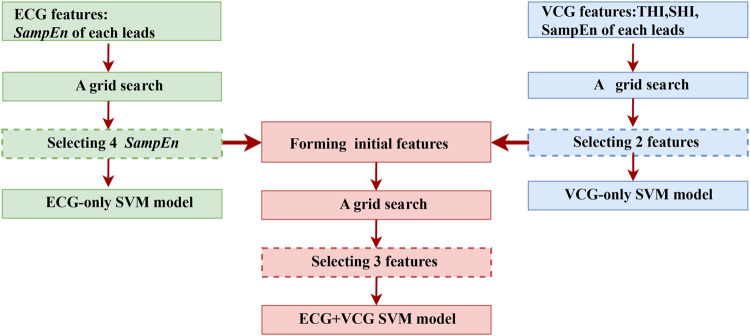
The flow chart of feature selection using a grid search on the training dataset

To minimize the number of the selected features and reduce the computational burden, a grid search was implemented to select the most influential features on the training dataset for each model, as shown in Figure 4. Firstly, all the features with an average accuracy of over 0.6 on the training dataset were selected for the ECG-only and VCG-only models, separately. Then, all possible permutations of the selected features were separately fed into the corresponding SVM models to pick out the most effective one for each model.

Regarding the ECG + VCG model, the selected ECG and VCG features rather than all of the ECG and VCG features (17 in total: 15 *SampEn*, *SHI*, *THI*) were fed into the SVM model. Subsequently, all possible permutations of the features with an average accuracy of over 0.6 were selected using the grid search to pick out the most useful one.

Finally, to verify that the grid search method can select the most useful features with the fewest features, for each model (i.e., ECG-only, VCG-only, ECG + VCG), the classification results using the features selected by the grid search were compared with those using the eigenvectors developed by the PCA algorithm, which is the commonest method for feature reduction (Alizadehsani et al., 2019). The eigenvector developed using the PCA algorithm for each model was titled as PCA-derived features. In the PCA algorithm, Minka’s maximum likelihood estimation was utilized to obtain the dimension of eigenvectors.

#### 2.4.3 Evaluation Criteria

We evaluated the classification performance of the constructed models using the area under the curve (AUC) of the receiver operating characteristic (ROC) curve and calculated the following metrics: accuracy, specificity, sensitivity, and F1 score according to the expressions in Eqs 12–15.

$$Accuracy=\frac{TP+TN}{TP+FP+FN+TN},$$ (12)

$$Specificity=\frac{TN}{TN+FP},$$ (13)

$$Sensivity=\frac{TP}{TP+FN},$$ (14)

$$F1=\frac{2TP}{2TP+FP+FN},$$ (15)

where *TP*, *TN*, *FP*, and *FN* represent the true positive, true negative, false positive, and false negative cases, respectively.

#### 2.4.4 Cross-Validation

Considering the limited size of our dataset, to avoid any overfitting caused by out-of-sample validation, we adopted a 5-fold cross-validation approach to evaluate the performance of the proposed models. The data was randomly divided into five groups, of which four groups were used for training and the last group was used as a test dataset for verification. This process was repeated five times, and the corresponding evaluation criteria for each calculation were recorded.

#### 2.4.5 Testing on a Third Independent Dataset

ECG parameters vary with ethnicity, age, gender, and region in different populations (Tan et al., 2016; Ching-Hui Sia, 2019). To further investigate the applicability and generalization ability of our final selected model, i.e., the ECG + VCG model employing (*S*_*I*_, *THI*, and *SHI*) for different populations, the testing was performed on a third independent dataset.

### 2.5 Statistical Analysis

The statistical analysis was performed on SPSS (Version 25.0, IBM Corp). Continuous values were presented as mean ± standard deviation (SD) for normally distributed data and median value (lower-upper quartiles) for non-normally distributed data. The categorical data was presented as numbers and percentages. The Kolmogorov-Smirnov test (*K-S* test) was used to check whether the data was normally distributed. Student’s *t* test (for normal distribution) or Wilcoxon signed rank test (for non-normal distribution) was deployed when appropriate. Statistical significance was defined as *p* values less than 0.05.

### 2.6 Development Environment

The experimental setup comprised an i7-8550u@1.98GHz CPU and 32GB of RAM. The data preprocessing and feature extraction were implemented using the (R2014; The MathWorks Inc., Natick, United States). The SVM models were designed and tested in Python 3.7 using Tensorflow 2.0 and the PCA algorithm.

## 3. Results

### 3.1 ECG-Only Model for Myocardial Ischemia Classification

**FIGURE 5:**
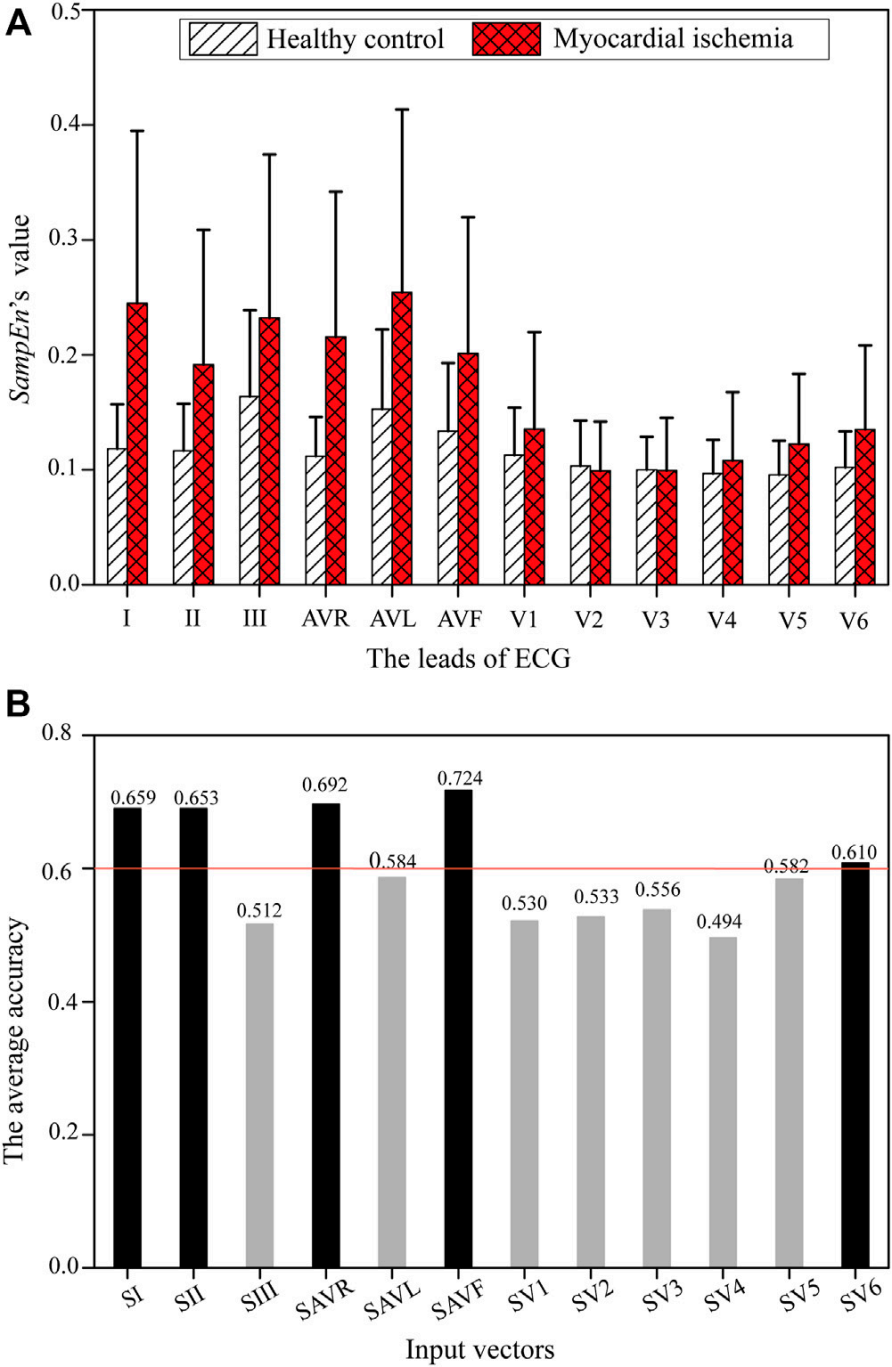
*SampEn* of each ECG lead from healthy controls and patients with myocardial ischemia. **(A)** *SampEn* of each ECG lead. **(B)** The average accuracy of an ECG-only model using each single Si on the training dataset.

The values of *SampEn* extracted from ECGs’ ST-T segments are shown in Figure 5A. The *SampEn’s* mean values of ischemic ECGs are obviously higher than those of healthy ones in most leads except *S*_*V2*_ and *S*_*V3*_.

Figure 5B shows the average training accuracy of the SVM models fed with *SampEn* of each ECG lead, i.e., *S*_*i*_. It can be observed that *S*_*I*_, *S*_*II*_, *S*_*AVR*_, *S*_*AVF*_, and *S*_*V6*_ are more influential than the remaining features, with an accuracy higher than 0.6. Table 2 lists two feature combinations selected using the grid search on the training database with the highest classification capabilities. (*S*_*I*_, *S*_*II*_, *S*_*AVF*_, *S*_*V6*_) outperforms (*S*_*I*_, *S*_*II*_, *S*_*AVR*_, *S*_*AVF*_, *S*_*V6*_) in respect of the major evaluation criteria except the training sensitivity. Thus, (*S*_*I*_, *S*_*II*_, *S*_*AVF*_, *S*_*V6*_) was selected as the input feature combination for the ECG-only model.

**TABLE 2:** Results of feature selection using the grid search for the ECG-only model on the training dataset

Input Vectors	Accuracy	Specificity	Sensitivity	F1 Score	AUC
(*S*_*I*_,*S*_*II*_,*S*_*AVF*_,*S*_*V6*_)	0.921 ± 0.004	0.911 ± 0.004	1.000 ± 0.000	0.953 ± 0.002	0.955 ± 0.002
(*S*_*I*_,*S*_*II*_,*S*_*AVR*_,*S*_*AVF*_,*S*_*V6*_)	0.889 ± 0.034	0.873 ± 0.009	1.000 ± 0.000	0.932 ± 0.006	0.936 ± 0.004

For validation, the grid search results were compared with those derived from PCA-derived features, as listed in Table 3. (*S*_*I*_, *S*_*II*_, *S*_*AVF*_, *S*_*V6*_) selected using the grid search outperformed PCA-derived features in all evaluation criteria. Therefore, (*S*_*I*_, *S*_*II*_, *S*_*AVF*_, *S*_*V6*_) was verified as the best candidate for the ECG-only model.

**TABLE 3:** Comparison of the classification effects of two different feature selection methods for the ECG-only model

Methods	Accuracy	Specificity	Sensitivity	F1 Score	AUC
Grid search	0.861 ± 0.033	0.876 ± 0.043	0.749 ± 0.131	0.916 ± 0.022	0.813 ± 0.057
PCA	0.766 ± 0.021	0.757 ± 0.022	0.829 ± 0.111	0.850 ± 0.015	0.793 ± 0.054

### 3.2 VCG-Only Model for Myocardial Ischemia Detection

**FIGURE 6:**
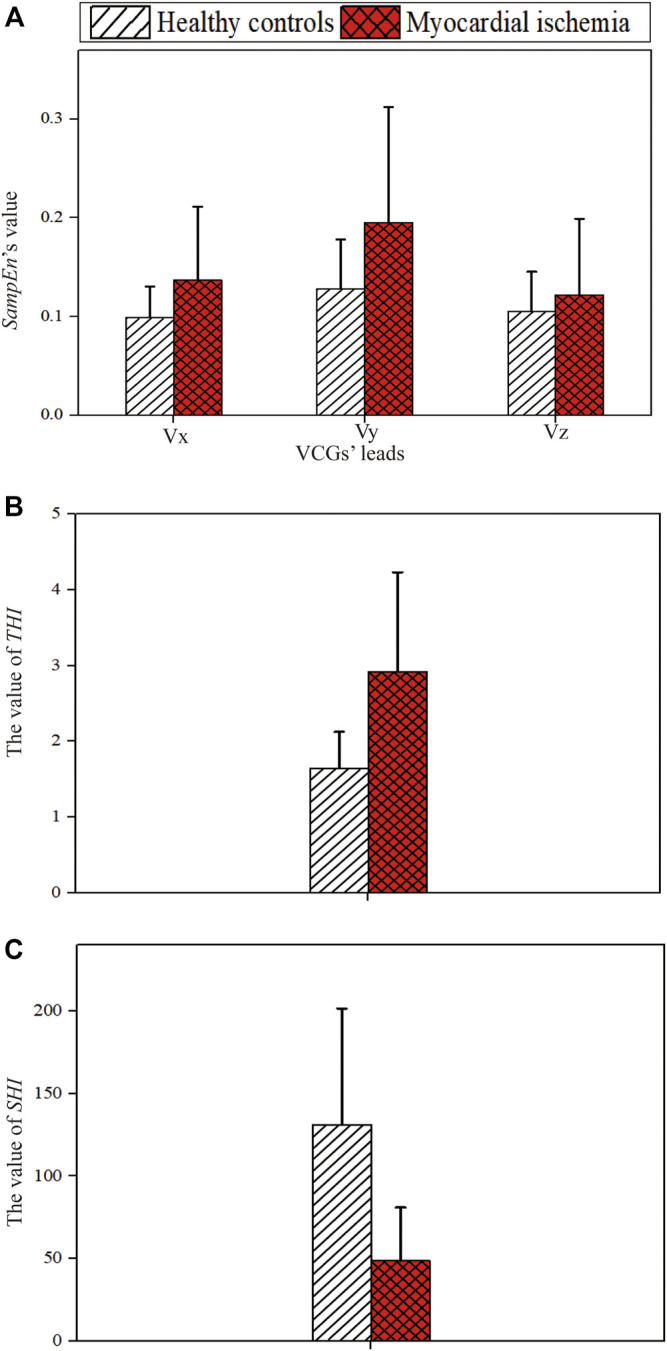
Deep features extracted from VCGs. **(A)** *SampEn*’*s* value. **(B)** THI. **(C)** SHI.

The values of *SampEn*, *SHI*, and *THI* extracted from VCGs’ ST-T segments are exhibited in Figure 6. Compared with healthy controls, the patients with myocardial ischemia show higher *SampEn* and *THI* (Figures 6A,B, respectively) but lower *SHI* (Figure 6C).

In the grid search, (*S*_*Vy*_, *THI*, *SHI*) and (*THI*, *SHI*) were selected for further comparison with their corresponding performance listed in Table 4, while the remaining feature permutations were excluded. On the training dataset, the SVM model utilizing (*THI*, *SHI*) outperformed that using (*S*_*Vy*_,*THI*, *SHI*) in major evaluation criteria except the training sensitivity and AUC. Therefore, (*THI*,*SHI*) was chosen as the candidate for the VCG-only model.

**TABLE 4:** Results of feature selection using the grid search for the VCG-only model on the training dataset

Input Vectors	Accuracy	Specificity	Sensitivity	F1 Score	AUC
(*THI*,*SHI*)	0.887 ± 0.005	0.889 ± 0.005	0.870 ± 0.018	0.932 ± 0.003	0.880 ± 0.010
(*S*_*vy*_,*THI*,*SHI*)	0.863 ± 0.016	0.856 ± 0.019	0.918 ± 0.018	0.917 ± 0.011	0.887 ± 0.012

In Table 5, the VCG-only model employing (*THI*, *SHI*) selected using the grid search outperforms the model with PCA-derived features in most items except specificity and AUC. Therefore, (*THI*, *SHI*) was verified as the input feature combination for the VCG-only model.

**TABLE 5:** Comparison of the classification effects of two different feature selection methods for the VCG-only model

Methods	Accuracy	Specificity	Sensitivity	F1 Score	AUC
Grid search	0.877 ± 0.034	0.884 ± 0.044	0.827 ± 0.073	0.926 ± 0.022	0.856 ± 0.029
PCA	0.870 ± 0.044	0.876 ± 0.046	0.829 ± 0.065	0.921 ± 0.029	0.921 ± 0.029

### 3.3 ECG + VCG Model for Myocardial Ischemia Diagnosis

To determine the best feature combination for the ECG + VCG model, all the selected features (i.e., *S*_*I*_, *S*_*II*_, *S*_*AVR*_, *S*_*V6*_, *SHI*, and *THI)* of the ECG-only and VCG-only models were included and selected using a grid search. In a grid search, (*S*_*I*_, *S*_*V6*_, *THI*, *SHI*) and (*S*_*I*_, *THI*, *SHI*) were picked up for further comparison with their corresponding modeling evaluation criteria listed in Table 6, while other permutations were ruled out. Table 6 demonstrates that (*S*_*I*_, *THI*, *SHI*) yields better performance than (*S*_*I*_, *S*_*V6*_, *THI*, *SHI*) on the training dataset. Thus, (*S*_*I*_, *THI*, *SHI*) were selected as the best candidates for the ECG + VCG model.

**TABLE 6:** Results of feature selection using the grid search for the ECG + VCG model on the training dataset

Input Vectors	Accuracy	Specificity	Sensitivity	F1 Score	AUC
(*S*_*I*_,*THI*,*SHI*)	0.907 ± 0.008	0.904 ± 0.008	0.923 ± 0.017	0.944 ± 0.005	0.913 ± 0.010
(*S*_*I*_,*S*_*V6*_,*THI*,*SHI*)	0.904 ± 0.007	0.902 ± 0.007	0.918 ± 0.024	0.942 ± 0.005	0.910 ± 0.014

In Table 7, the ECG + VCG model employing (*S*_*I*_, *THI*, *SHI*) selected by a grid search outperforms that employing PCA-derived features in all evaluation criteria. Therefore, (*S*_*I*_, *THI*, *SHI*) were validated as the optimal feature combinations for the ECG + VCG model.

**TABLE 7:** Comparison of the classification effects of two different feature selection methods for the VCG + ECG model

Methods	Accuracy	Specificity	Sensitivity	F1 Score	AUC
Grid search	0.903 ± 0.040	0.903 ± 0.043	0.905 ± 0.086	0.942 ± 0.025	0.904 ± 0.049
PCA	0.894 ± 0.039	0.898 ± 0.046	0.865 ± 0.046	0.936 ± 0.025	0.882 ± 0.028

### 3.4 Comparison Among ECG-Only, VCG-Only, and ECG + VCG Models

**FIGURE 7:**
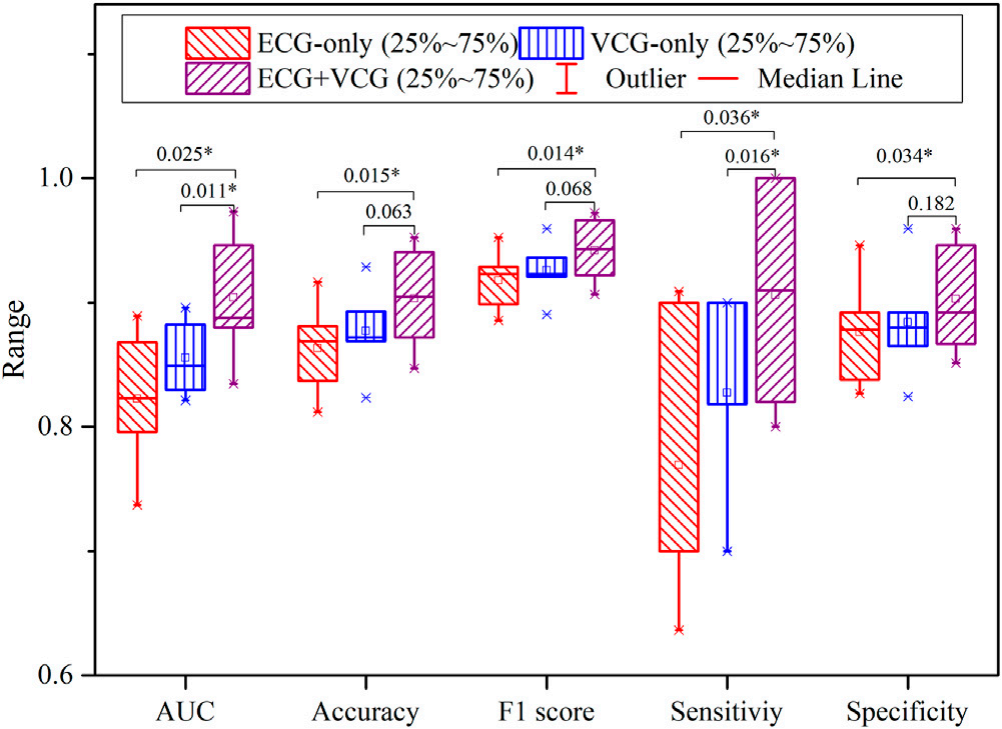
The boxplots of the accuracy, specificity, sensitivity, F1 score, and AUC for myocardial ischemia detection correspond to the ECG-only, VCG-only, and ECG + VCG models.

Figure 7 shows the quantitative comparison of evaluation criteria among ECG-only, VCG-only, and ECG + VCG models. The ECG + VCG model achieves higher median and mean values for the evaluation criteria than any of the remaining models. The Student’s *t*-tests show that the ECG + VCG model is significantly better than the ECG-only model in all evaluation criteria (*p* < 0.05 for all). Meanwhile, it is significantly superior to the VCG-only model in terms of sensitivity and AUC (*p* < 0.05 for both). Therefore, the ECG + VCG model employing (*S*_*I*_, *THI*, *SHI*) was the optimal one for myocardial ischemia detection.

### 3.5 Testing of the Selected Model on a Third Independent Dataset

On the third independent dataset, the testing results of the selected ECG + VCG model employing (*S*_*I*_, *THI*, *SHI*) were 0.790 accuracy, 0.764 sensitivity, 0.865 specificity, 0.843 F1 score, and 0.814 AUC.

## 4. Discussion

### 4.1 Summary of Results: In Comparison With Existing Studies

In this work, we developed a reliable ECG + VCG SVM model for myocardial ischemia detection using the features extracted from VCGs’ and ECGs’ ST-T segments. The classification effectiveness of three models was comprehensively compared across five evaluation criteria.

The comparison of our model against state-of-the-art myocardial ischemia detection approaches is listed in Table 8. Our model yields better effectiveness by using a much lower number of features but longer ECG and VCG signals. The major reason behind this is that the comprehensive utilization of ECGs’ and VCGs’ ST-T segments could capture more ischemia-induced temporal and spatial changes. Specifically, ischemia-related beat-to-beat changes in ST-T segments were measured by the temporal and spatial features used in this work, while beat-based (Correa et al., 2014; Aranda Hernandez et al., 2018; Braun et al., 2020) ML models only utilized the changes in a single heartbeat. Beat-to-beat changes in the T wave or ST segment result from the increase in ischemia-induced repolarization dispersion between ischemic and healthy regions as well as between different ischemic regions (Arini et al., 2014). *SHI* was extracted to evaluate the beat-to-beat changes of VCGs in 3D space. Besides, ischemia-induced temporal changes can be reflected in the VCG waveform and the time delays among ECG leads (Correa et al., 2014). Therefore, the temporal characteristics of beat-to-beat changes in ST-T segments were assessed by VCGs’ *THI* and ECGs’ *SampEn* simultaneously*.* Besides, longer signals used in this work could enhance the total scheme efficiency and overall accuracy (Hussein et al., 2021; Li et al., 2021).

**TABLE 8:** Comparison of the proposed model against existing approaches on myocardial ischemia detection

Authors	Year	Algorithm	Cohorts	Signals	Num. of Features	Performance
Kors et al. (1992)	1992	Decision tree	1,220 subjects from CSE database	10 s VCGs and ECGs	Unknown	*Overall Acc:*
						84.3
Dehnavi et al. (2011)	2011	Forward neural networks	60 ischemic patients, 10 healthy controls	3 s VCGs	22	*Sen*:70
						*Spec*:86
Correa et al. (2013)	2013	Linear discriminant analysis	80 ischemic patients in MI from STAFF III database, 52 healthy controls from PTB database	VCG beats	8	*Sen*:88.5
						*Spec*: 92.1
Correa et al., (2014)	2014	Linear discriminant analysis	80 ischemic patients before (control) and during PTCA from STAFF III database	VCG	4 out of 12	Sen: 90.5
				Beats		Spec:92.5
Aranda Hernandez et al. (2018)	2018	The gradient boosting method	98 ischemic patients before (control) and during PTCA from STAFF III database	10 s VCGs	7 out of 328	*Sen*: 89.6
						*Spec*:82.7
Braun et al. (2020)	2019	Five feed forward neural networks.	406 ischemic patients,189 non-CAD patients	VCG beats	27 out of 2,320	For female:
						*Sen*: 90.2 ± 4.2
						*Spec*:74.4 ± 9.8
						Overall *Acc*:
						82.5 ± 6.4
This work	2021	SVM	377 ischemic patients, 52 healthy controls from PTB database	20 s ECGs and VCGs	3	*Acc*: 0.90 ± 0.04
						*Sen*: 0.90 ± 0.04
						*Spec*: 0.90 ± 0.08

abbreviations: *Acc*: accuracy. *Sen*: sensitivity. *Spec*: specificity. CSE, database: Common Standards for Quantitative Electrocardiography database. PTCA: percutaneous transluminal coronary angiography.

In some existing studies, patients with myocardial infarction were selected from the STAFF III database as positive samples of ischemia (Correa et al., 2013; Correa et al., 2014; Aranda Hernandez et al., 2018), in which the ischemic changes in ECG waveform are more obvious than asymptomatic patients. In comparison, we included ischemic patients with CAD but not limited to symptomatic ones with myocardial infarction, which provides a better understanding of ischemia instead of focusing on myocardial infarction. Moreover, the VCGs from the same patient with myocardial infarction before and during the percutaneous transluminal coronary angiography (PTCA) procedure were selected from the STAFF III database as the corresponding negative and positive samples (Correa et al., 2014; Aranda Hernandez et al., 2018). In contrast, our patients and controls were recruited separately, where the effect of clinical intervention could be excluded to better reflect the ischemic changes.

Compared with state-of-the-art myocardial ischemia detection approaches, our final selected model was tested on a third independent database rather than a testing dataset separated from the same cohort. ECG parameters depend on physiological factors including ethnicity, sex, age, and body size (Tan et al., 2016; Ching-Hui Sia, 2019). As a result, ECG-based screening of CAD is influenced by a myriad of factors, including demographics, anthropometrics, level of physical fitness, and population-specific reference ranges among distinct population groups (Ching-Hui Sia, 2019). Therefore, testing on a third independent database can comprehensively evaluate the algorithmic applicability and generalization ability for different populations. As far as we know, we tried the testing in different populations for the first time. Despite the inconsistent data distribution and the heterogeneity between cohorts, the major evaluation criteria are above 0.8, except for the sensitivity and accuracy, which demonstrated that our proposed model provides the possibility for applications in different populations.

### 4.2 Importance of VCG in Detecting Myocardial Ischemia

Our ECG + VCG model outperforms the ECG-only and VCG-only ones for myocardial ischemia detection, which is in accordance with existing studies (Lee et al., 1968; Häggmark et al., 2008; Correa et al., 2016). The ECG measurements are based on the lead theory, which assumes that cardiac electrophysiological activities form a heart vector which moves periodically in 3D space in cardiac cycles, forming VCG loops. Ischemia-induced repolarization dispersion can change the temporal and spatial properties of the heart vector. Thus, the ST vector is called the “ischemia vector” due to its sensitivity to myocardial ischemia. VCG could reflect the dynamics of repolarization abnormalities (Hasan and Abbott, 2016). Both ECG and VCG signals have ischemia-related temporal changes, while VCG uniquely shows the spatial changes.

VCG is a recurring, near-periodic pattern of cardiac dynamics and represents the trajectory of the tip of heart vectors in 3D space. From VCG which reflects both the magnitude and direction of the heart vector, the dysfunction of the ischemia-related back region of the heart can be detected. In contrast, ECG is the secondary projection of VCG loops on the lead axis (Okamoto et al., 1982), which only reflects the magnitude but not the orientation of the heart vector (Hasan et al., 2012). A particular ECG lead describes the heart vector from a fixed direction, making it difficult to detect electrical activity in some areas of the heart. For example, 12-lead ECG has limited sensitivity in detecting an acute posterior injury pattern during circumflex coronary artery ischemia (Khaw et al., 1999). Therefore, VCG plays a key role in improving the accuracy in detecting myocardial ischemia.

### 4.3 Relationship Between Features and Myocardial Ischemia

In this study, *S*_*I*_, *THI*, and *SHI* were selected as the most crucial features for the ECG + VCG model. These features can reflect the heterogeneous repolarization process resulting from the decrease in conduction velocity and the duration of action potential leads (Janse and Wit, 1989) during myocardial ischemia.

*SampEn* extracted from ischemic ST-T segments is higher than that from healthy ones in most leads, as shown in Figure 5A; Figure 6A. The heterogeneous repolarization is reflected in the ECG via the changes in ST-segment and T-wave (Correa et al., 2014), such as ST segment elevation or depression and T-wave changes (e.g., inverted T wave, biphasic T wave, or high-tip T wave). Therefore, these ischemia-related changes increase the complexity of beat-to-beat ST-T segments, leading to a higher *SampEn*. It is demonstrated that ST segments induced by myocardial ischemia have relatively large morphological variability (Wei et al., 2012). The average *SampEn* obtained from ischemic ST segments of ECGs was found to be higher than that of healthy subjects (Rabbani et al., 2011). *SampEn* extracted from the filtered ECGs could reflect ischemia-induced myocardial infarction (Liu et al., 2019). Therefore, *SampEn* is a reliable feature of myocardial ischemia.

The results in Figures 6B,C and Table 5 suggest that *SHI* and *THI* calculated from VCGs’ ST-T segments reflect the heterogeneity of ventricular repolarization induced by ischemia. Usually, the heterogeneous repolarization is reflected in the VCG by the changes in the QRS loop, T-wave vector, and ST vector (Correa et al., 2014). Spatial TT′ angle and beat-to-beat variability in T-loop roundness represent intrinsic measures of beat-to-beat repolarization ability (Feeny and Tereshchenko, 2016). Compromised hearts have irregular and distorted T-loops, whilst healthy ones have smooth planar loops. Myocardial ischemia changes the T vector angle and T loop morphology. Meanwhile, the spatial orientations and magnitudes of ST vectors were not stable (ter Haar et al., 2013). Therefore, features of VCGs’ ST-T segments can be indicators of myocardial ischemia (Correa et al., 2014; Dong et al., 2018). ST-T interval characterizations have significant differences before starting and during the PTCA procedure in patients with acute myocardial ischemia (Correa et al., 2014). We hypothesize that beat-to-beat changes in T vector and ST vector make ischemic trajectories of VCGs’ ST-T segments more chaotic with more perturbations compared with healthy ones. *SHI* and *THI* can capture the ischemia-related chaotic spatial and temporal characteristics of VCGs and therefore distinguish ischemic and normal subjects (Deng et al., 2017).

### 4.4 Advantages, Limitations, and Future Directions

Our proposed model affords the possibility of noninvasive detection of myocardial ischemia in different populations. It could be implemented in ECG acquisition systems with VCG mathematically synthesized from standard 12-lead ECG. There will be no extra workload for operators, since feature extraction and classification algorithms are automatic. Compared with PCA-derived features calculated from all features using a linear transform, our model is based on the minimal number of features selected using a grid search, which is achievable on wearable devices where the computational resources are limited. Therefore, it could become a practical, easy-to-accept, and cost-effective tool for myocardial ischemia detection in various application scenarios, including routine community screening, older people’s homes, and daily monitoring using wearable ECG sensors. The results can provide an important reference for clinicians on the early diagnosis of CAD.

There are some limitations to this work. First, the number of samples, specifically negative samples, is relatively small. Secondly, the evaluation criteria of our final selected model on a third independent dataset are lower than those of other state-of-the-art algorithms since ECGs from the training and testing datasets were collected from different populations with different acquisition equipment. Thus, the difference in physiological characteristics may affect the results. Thirdly, we focused on the electrophysiological features, while other clinical examination results were not included in our model. In future studies, the feature extraction from 10 s ECGs can be explored to enable daily clinical use. The combination of ECG features and clinico-radiological parameters can be deployed to achieve higher accuracy in ischemia detection. A large-scale mixed database of multicenter datasets in different populations can be built to further verify our conclusions and improve the accuracy among different populations.

## 5. Conclusion

The ECG + VCG model can outperform the ECG-only and VCG-only models in identifying myocardial ischemia using only three features (*S*_*I*_, *THI*, and *SHI*) extracted from VCGs’ and ECGs’ ST-T segments, providing a potential tool for non-invasive detection of myocardial ischemia.

## Data Availability

The raw data supporting the conclusion of this article will be made available by the authors without undue reservation.
