# Supplementary material for: Reliable Detection of Myocardial Ischemia Using Machine Learning Based on Temporal-Spatial Characteristics of Electrocardiogram and Vectorcardiogram
Source: Front Physiol. 2022 May 30;13:854191. doi: 10.3389/fphys.2022.854191 (PMC9192098; doi:10.3389/fphys.2022.854191)
Supplement: Supplementary file 1 [file DataSheet1.docx]

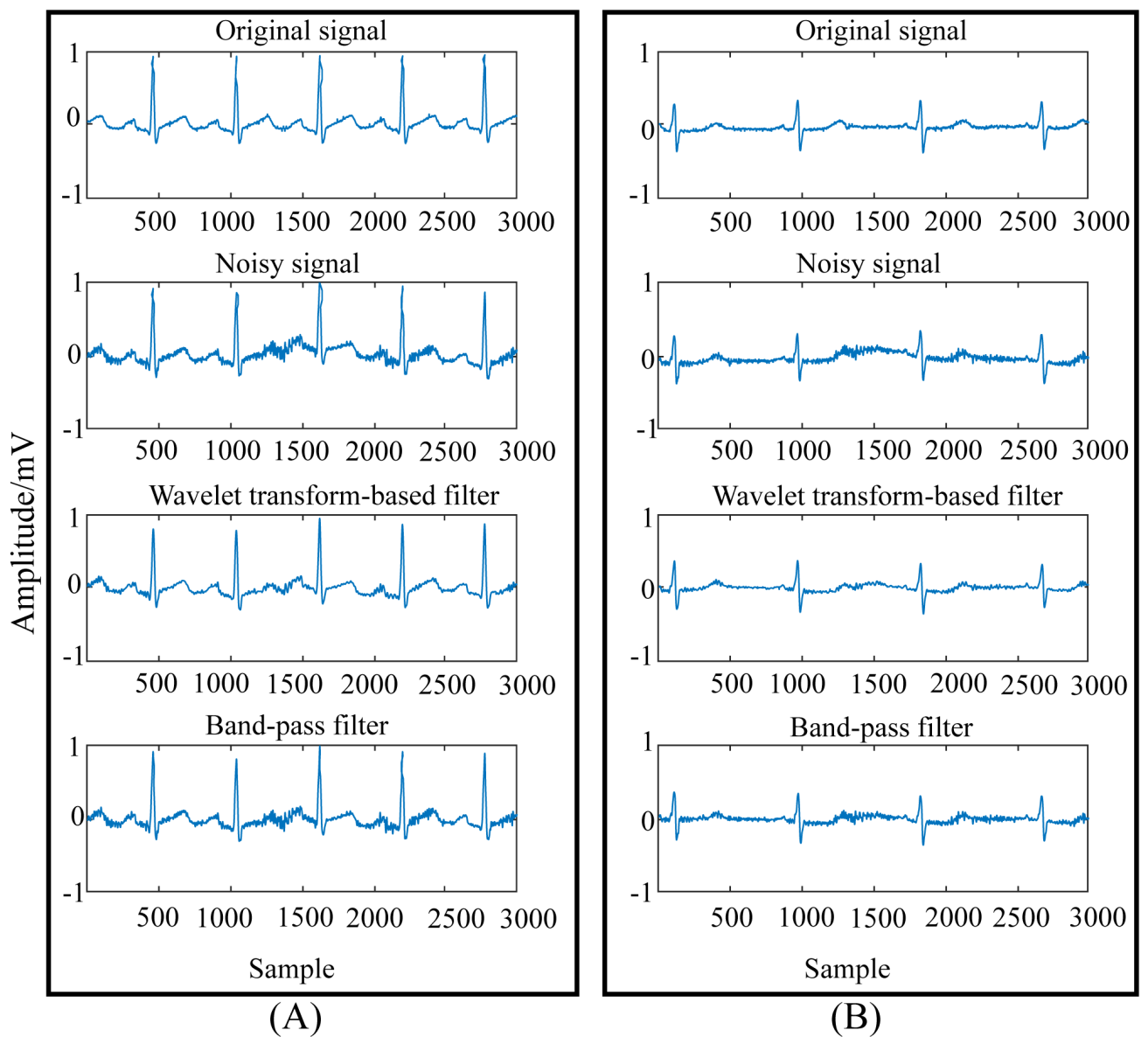


Supplementary Figure 1 Qualitative comparison of the effects of eliminating electromyogram noise (muscle artifact) between our proposed method (the third row in both columns) and a Butterworth band-pass filter (the fourth row) with 0.67 Hz low-frequency cutoff and 150 Hz high-frequency cutoff. (A) ECG recording of a healthy control from PTB database. (B) ECG recording of a patient with myocardial infraction from PTB database.

The noisy ECG recordings were created by adding calibrated amounts of electromyogram noise from MIT-BIH Noise Stress Test Database (nstdb) (https://physionet.org/content/mitdb/1.0.0/) to original ECG recordings with (signal-noise-rate (SNR) = 5dB). It can be observed that the level of residual noise is much lower when preprocessed using our proposed method compared with the band-pass filter.
